# In vitro response pattern of monocytes after tmTNF reverse signaling predicts response to anti-TNF therapy in rheumatoid arthritis

**DOI:** 10.1186/s12967-015-0620-z

**Published:** 2015-08-07

**Authors:** Undine Meusch, Marco Krasselt, Manuela Rossol, Christoph Baerwald, Maria Klingner, Ulf Wagner

**Affiliations:** Rheumatology Unit, Department of Internal Medicine, University of Leipzig, Liebigstr. 20, 04103 Leipzig, Germany

**Keywords:** Rheumatoid arthritis, TNF inhibition, Monocytes, Cytokines, tmTNF reverse signaling, Prediction, Therapy response

## Abstract

**Background:**

Treatment with TNF inhibitors is very efficient in the majority of the patients with rheumatoid arthritis (RA), but it does not achieve a sufficient treatment response in 40–50% of the cases. Goal of the study was to assess functional ex vivo-tests of RA monocytes as prognostic parameters of the subsequent treatment response.

**Methods:**

20 anti-TNF naïve RA patients were enrolled in a prospective, open-label trial, and Etanercept therapy was initiated. Prior to treatment, reverse signaling was induced in peripheral blood monocytes by tmTNF crosslinking via TNFR2:Ig construct Etanercept in a standardized ex vivo-assay. Released cytokine and cytokine receptor concentrations were determined as parameters of the monocyte response.

**Results:**

Crosslinking of tmTNF and consecutive reverse signaling led to production of pro- and anti-inflammatory cytokines and of soluble cytokine decoy receptors such as sTNFR1 and sIL-1R2. Several of the measured concentrations were found to correlate with the treatment response according to the EULAR criteria. The correlation was most pronounced in sTNFR1 concentrations (r = −0.657, p = 0.0031), which also predicted a good clinical response with the highest sensitivity and specificity according to EULAR criteria.

**Conclusions:**

Herein we propose that the tmTNF crosslinking-triggered shedding of soluble decoy receptors and production of anti-inflammatory cytokines could contribute to the clinical efficacy of TNF inhibitors, and that in vitro quantification of this secretion by RA monocytes prior to treatment can be used to predict the clinical response. Further development of such standardized tests could be a step towards personalized medicine by providing rheumatologists with a rational choice for first line biological therapy in patients with RA.

## Background

The clinical efficacy of specific cytokine inhibitors in rheumatoid arthritis (RA) reveals the pivotal role of predominantly monocytic cytokines like IL-1β, TNF or IL-6 in the pathogenesis of the disease. The relevance of the circulating monocyte pool is highlighted by several pathological subpopulations including CD14^bright^ CD16^+^ as well as CD14^bright^ CD56^+^ cells [1, 2]. In addition to those pathological subpopulations, the global monocyte pool in RA is characterized by increased cell surface expression of transmembrane TNF (tmTNF), which is not detectable in healthy controls [3].

We and others have extensively investigated the cellular signaling pathways initiated by tmTNF ligation with soluble tmTNF ligands like anti-TNF antibodies or TNFR2:Ig construct Etanercept [3–7]. This so-called reverse signaling inhibits the intrinsic NFkappaB activation and IL-1β secretion characteristic for RA monocytes, and induces apoptosis [3]. Contrary to expectations, the rate of apoptosis induced by reverse signaling is not related to the therapeutic efficacy of TNF inhibitors [8].

In Crohn’s disease, anti-TNF antibodies are also binding to tmTNF positive myelomonocytic cells [9]. Patients with high numbers of tmTNF^+^ immune cells have higher response rates to anti-TNF treatment due to higher rate of apoptosis [10], which is in contrast to our findings in patients with RA.

In RA, tmTNF ligation by soluble anti-TNF antibodies induces not only apoptosis, but also shedding of the soluble cytokine decoy receptors IL-1sRI and IL-1sRII. Secretion levels of IL-1sRII in vitro are linked to a good response to anti-TNF blockade [8]. This production is significantly enhanced when tmTNF molecules are not merely ligated by a soluble ligand, but are cross-linked by surface immobilized antibodies [7]. Importantly, in such scenario, induction of apoptosis does not occur.

To investigate the response of RA monocytes to such tmTNF crosslinking by a ligating molecule immobilized on plastic or cell surfaces, a standardized in vitro assay was established. Goal of the study was to investigate tmTNF crosslinking-induced cytokine production as a potential prognostic parameter for the therapeutic response to Etanercept. The results show that the crosslinking-induced concentrations of anti-inflammatory cytokines and soluble decoy receptors can distinguish responders from non-responders prior to initiation of therapy.

## Methods

### Patients and study design

Details of study design, inclusion criteria, and clinical documentation performed have been reported previously [8]. The design of the clinical study has been approved by the ethics committee of the University of Leipzig, and informed consent was obtained from each patient before enrolment into the study. A total of 33 consecutive, anti-TNF naïve patients with RA according to the revised criteria of the American College of Rheumatology (ACR) [11], who were assessed by their rheumatologists to require treatment with Etanercept based on their clinical status, were screened for the study. 13 of them could not be initiated on anti-TNF treatment due to subsequently discovered various contraindications, newly diagnosed co-morbidities, requirement of surgery or withdrawal of patient’s consent. In 20 patients, Etanercept treatment was started.

At baseline, 80% (16 patients) were treated with conventional DMARDs, given either as monotherapy or in combination, and 20% received glucocorticoids only. All patients were treated with non-steroidal anti-inflammatory drugs (NSAIDs) for symptomatic relief. During the study, two patients withdrew their consents before efficacy could be assessed.

In the final cohort of 18 patients, mean age was 53 years, and the mean disease duration was 4 years: 65% of the patients were RF IgM-seropositive, and 80% had anti-CCP antibodies. None of the patients showed any clinical sign of an infection at baseline visit.

Blood samples for monocyte isolation were taken at baseline, week 4, week 12 and week 24. Each visit included a physical examination, routine laboratory tests and assessment of the patient’s global health in order to determine the current disease activity (Disease Activity Score 28 joints, DAS28). The mean DAS28 of all patients before initiating treatment was 4.69, at week 4 3.51, at week 12 3.17 and after 24 weeks of therapy 2.84.

After 4 weeks of therapy, 80% of the patients reached a response as defined by EULAR [12] with 31.25% achieving a good clinical response and 68.75% achieving a moderate response. Response rates increased during the study and reached a good response in 56.25% of all responders after 6 months of treatment.

A good response according to EULAR is defined as an improvement in DAS28 >1.2 (present DAS28 ≤3.2), a moderate response as an improvement between 0.6 and 1.2 (present DAS28 ≤5.1) or >1.2 (present DAS28 >3.2). Improvements ≤0.6 or between 0.6 and 1.2 (present DAS28 >5.1) are considered as no response.

### Monocyte isolation, cell culture and stimulation

Monocytes from peripheral blood were separated as previously described [7]. 2 × 10^5^ monocytes per 200 µl were incubated in RPMI 1640 supplemented with 5% human AB serum (heat-inactivated). Stimulation of cells was carried out either with 100 µg/ml rituximab (Roche, Basel, Switzerland) as an IgG control or with TNFR2:Ig construct Etanercept (100 µg/ml, Pfizer, New York, NY, USA) for 16 h, either as a soluble or a plate-bound molecule, as indicated for individual experiments.

### Flow cytometry

After separating monocytes, cells were stained with fluorescence-bound antibodies for CD14, TNFR1, TNFR2 and CD54 (R&D Systems, Minneapolis, MN, USA) and analyzed on a FACSCalibur system.

### Cytokine detection

Cytokine levels in cell culture supernatants were measured with cytometric bead arrays (CBA) or enzyme-linked immunosorbent assay (ELISA) technique (BD biosciences, Franklin Lakes, NJ, USA) according to the manufacturer’s protocol.

### Statistics

All analyses were conducted using GraphPad PRISM Version 6 (GraphPad Software Inc., San Diego, CA, USA). Normality test was performed prior to all comparisons. To assess statistical significance, Student’s *t* test was used on normal distributions. Otherwise, Mann–Whitney rank sum test was performed. A statistical difference was considered when the p value was less than 0.05.

Correlation between two parameters was analyzed with either Pearson’s product–moment correlation (for normally distributed data) or Spearman (for data not normally distributed).

## Results

### Increased frequencies of TNFR1+ monocytes and decreased monocytic CD54 expression are associated with a good therapeutic response to TNF inhibition

**Fig. 1:**
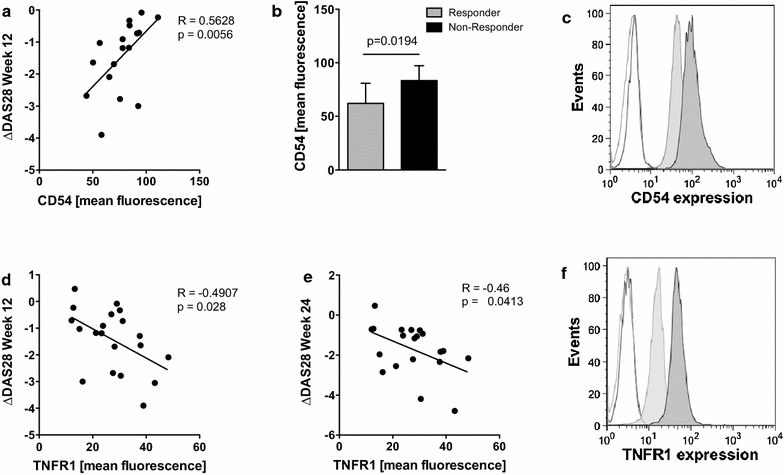
Monocytic surface expression of TNFR1 and CD54 are linked to anti-TNF treatment response. **a** Scatterplot showing the correlation of CD54 expression on ex vivo separated RA-monocytes with the decrease in the patients’ DAS28 (ΔDAS28) after 12 weeks (n = 18). Receptor expression is given as mean fluorescence intensity as determined by FACS. **b** Bar graph depicts mean and SEM of CD54 expression (mean fluorescence) in the study cohort on monocytes in patients with a good EULAR response (mean 62.1 ± 8.4) and non-responders (mean 83.48 ± 3.99), respectively. **c** Cell surface expression of CD54 was determined by flow cytometry on freshly isolated monocytes. Shown are representative histograms from one patient with a good response (*light grey curve*) and from a non-responder (*dark grey curve*) in comparison to the isotype controls (*white curve*; *dark grey line* for non-responder, *light grey line* for good responder). **d**, **e** Scatterplot showing the correlation between TNFR1 expression determined ex vivo on RA monocytes and the decrease in DAS28 (ΔDAS28) after 12 weeks (n = 17) (**d**) and 24 weeks (n = 18) (**e**). TNFR1 expression was determined by mean fluorescence intensity. **f** Cell surface expression of TNFR1 on freshly isolated monocytes. Shown are representative histograms from one patient with a good response (*dark grey filled curve*) and from a non-responder (*light grey filled curve*) in comparison to the isotype controls (*dark grey line* for good responder, *light grey line* for non-responder). Non-responders are defined as patients achieving only a moderate or no response. DAS28, disease activity score 28.

We found a significant correlation between the mean expression of Intercellular Adhesion Molecule 1 (ICAM-1, CD54) and the DAS28 response (ΔDAS28) at week 12, and significantly lower values in EULAR responders (Fig. 1a, b, representative histograms in Fig. 1c). The frequency of monocytes positive for CD54, however, did not differ between responders and non-responders (data not shown). In addition, the mean level of TNFR1 expression on monocytes correlated significantly with the DAS28 response after 12 weeks (r = −0.4907, p = 0.028, Fig. 1d) and after 6 months (r = −0.46, p = 0.041, Fig. 1e, representative histograms in Fig. 1f). No such correlation was observed for TNFR2 expression (data not shown).

### Cytokine decoy receptor levels induced by tmTNF crosslinking predict subsequent responses to anti-TNF therapy

**Fig. 2:**
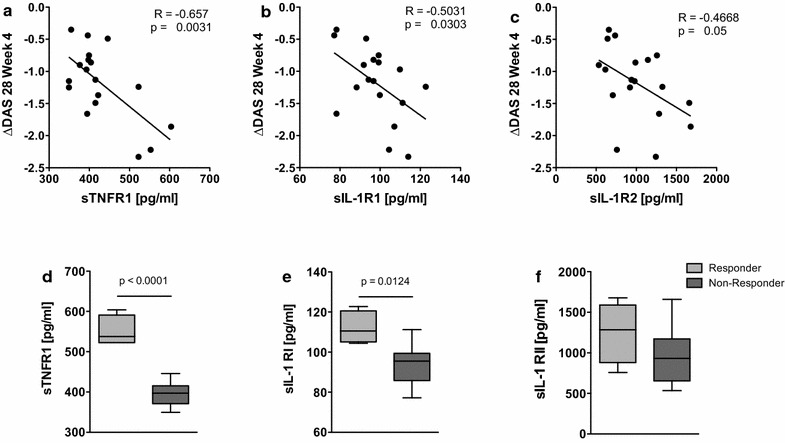
tmTNF crosslinking-induced cytokine decoy receptor levels are associated with a good response to anti-TNF treatment. **a**–**c** Scatterplots depict the correlation of tmTNF crosslinking induced monocytic secretion of sTNFR1 (**a**), sIL-1R1 (**b**) and sIL-1R2 (**c**) with the decrease in the patients' DAS28 (ΔDAS28) after 4 weeks of anti-TNF therapy. Each *dot* represents one patient (n = 18). **d**–**f** Box plots depict the concentrations of the tmTNF crosslinking-induced cytokine decoy receptors sTNFR1 (**d**), sIL-1R1 (**e**) and sIL-1R2 (**f**) in patients with a good response according to the EULAR response criteria (responder, *light grey bars*, n = 4) compared to patients achieving only a moderate or no response (non-responder, *dark grey bars*, n = 14). Levels of significance as indicated.

In vivo, the effects of the pro-inflammatory monocytic cytokines TNF and IL-1β are counterbalanced by soluble cytokine decoy receptors released from the cell surface by shedding. tmTNF crosslinking with the TNFR2:Ig construct Etanercept was found to induce the release of several cytokine decoy receptors in RA monocytes. The tmTNF crosslinking-induced concentrations of sTNFR1, sIL-1R1 and sIL-1R2 at baseline were all found to correlate with the DAS28 response after 4 weeks of observation (Fig. 2a–c). The strongest correlation with ΔDAS28 was found in sTNFR1 (r = −0.657, p = 0.0031). tmTNF crosslinking-induced shedding of the decoy receptors was significantly higher in patients with a good EULAR response than in those with moderate or no response (Fig. 2d–f). In contrast to the effects of tmTNF crosslinking with the TNFR2:Ig construct Etanercept, crosslinking with therapeutic anti-TNF antibodies did not induce cytokine responses (data not shown).

### Secretion of cytokines induced by tmTNF reverse signaling and crosslinking is predictive of the subsequent therapeutic response

**Fig. 3:**
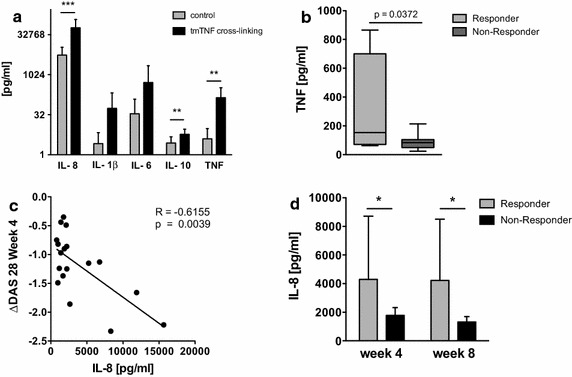
Reverse signaling and tmTNF crosslinking-induced cytokine secretion is higher in responders to TNF inhibitors. **a** Bar graph shows concentrations of tmTNF crosslinking-induced secretion of the indicated cytokines (*black bars*) and concentrations found in control cultures containing an irrelevant, plate-bound antibody (*grey bars*, n = 18). Levels of significance: *p < 0.05, **p < 0.01, ***p < 0.001. **b** Box plots depict the concentrations of the tmTNF crosslinking-induced TNF secretion in patients with a good response according to the EULAR response criteria (responder, n = 4) compared to patients achieving only a moderate or no response (non-responder, n = 14). **c** Scatterplot depicts correlation of concentrations of reverse signaling-induced IL-8 with the decrease in DAS28 (ΔDAS28) after 4 weeks of therapy. IL-8 was measured by ELISA (n = 18). **d** Bar graph depicts reverse signaling-induced IL-8 concentrations in patients with any EULAR response (*grey bar*, n = 15) compared to those with no response at all (*black bar*, n = 3) at the given time points. Levels of significance: *p < 0.05, **p < 0.01, ***p < 0.001.

Transmembrane TNF crosslinking in vitro for 16 h triggered the production of several cytokines, while incubation with a plate-bound irrelevant control antibody induced only significantly lower concentration (Fig. 3a). The tmTNF crosslinking-induced secretion of TNF was significantly higher in patients who achieved a good EULAR response after 4 weeks of treatment (Fig. 3b).

Secretion levels of IL-8, when triggered only by incubation with soluble tmTNF ligand, were also found to correlate with the ΔDAS28 response observed after 4 weeks of therapy (r = −0.6155, p = 0.0039, Fig. 3c). Moreover, monocytes from patients achieving good or moderate responses according to the EULAR criteria responded with significantly higher IL-8 secretion upon crosslinking-independent reverse signaling at baseline than monocytes from non-responders (Fig. 3d).

Longitudinal analysis of tmTNF crosslinking induced cytokine secretion revealed a significant reduction in the concentrations of TNF (p < 0.0001) and IL-8 (p = 0.0311) in the supernatant, but no difference in any of the other cytokines or soluble receptors analyzed (data not shown).

### Secretion of IL-10 induced by tmTNF crosslinking in RA monocytes predicts the subsequent therapeutic response

**Fig. 4:**
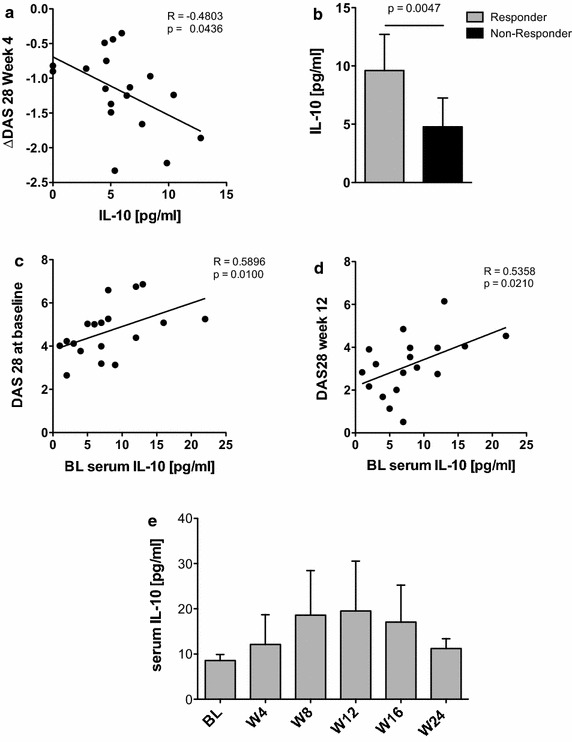
tmTNF crosslinking-induced IL-10 secretion and serum IL-10 concentrations are associated with clinical response and disease activity, respectively. **a** Scatterplot depicts correlation of tmTNF crosslinking-induced IL-10 with the decrease in DAS28 (ΔDAS28) after 4 weeks of therapy (n = 18). **b** Bar graph depicts tmTNF crosslinking-induced IL-10 concentrations in patients with a good EULAR response (responder, n = 4) compared to those with a moderate or no response (non-responder, n = 14) after 4 weeks of anti-TNF therapy. **c**, **d** Scatterplots showing the correlation of serum concentrations of IL-10 determined in RA patients at baseline (n = 18) with the disease activity (DAS28) at baseline (**c**) and after 12 weeks of anti-TNF therapy (**d**). **e** Longitudinally determined serum concentrations of IL-10 in RA patients under anti-TNF treatment. Levels of significance as indicated. *BL* baseline, *W* week.

Concentrations of the anti-inflammatory cytokine IL-10 induced by tmTNF crosslinking were also found to correlate with the DAS28 response after 4 weeks (r = −0.4803, p = 0.0436, Fig. 4a) and to be higher in patients with a good EULAR response after 4 weeks of treatment (Fig. 4b).

In order to investigate a possible link between IL-10 and disease activity in vivo, we determined serum concentrations of the cytokine at baseline and found them to be significantly related to disease activity at baseline (Fig. 4c) and week 12 (Fig. 4d). This correlation was lost later on in the longitudinal analysis during the 6 months of follow-up under anti-TNF therapy. Interestingly, we also found that patients under anti-TNF treatment show a continuous increase of serum concentration of IL-10 (Fig. 4e), which might contribute to clinical efficacy of the treatment.

### Receiver operating characteristic (ROC) analysis of tmTNF crosslinking-induced parameters

**Fig. 5:**
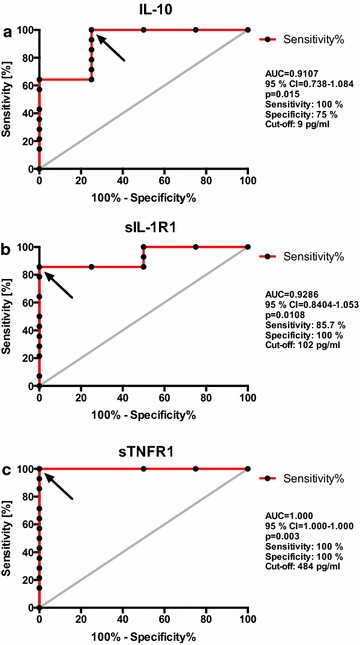
ROC curve analysis for predicting the response to TNF inhibiting agents. The diagnostic accuracy [frequency (in %) of true positives = sensitivity and of false positives = 100% − specificity] is presented for the tmTNF crosslinking-induced concentrations of IL-10 (**a**), sIL-1R1 (**b**) and sTNFR1 (**c**). Each *dot* represents one patient (n = 18). Area under the curve (AUC), confidence intervals (95% CI) and level of significance are given for each analysis. Sensitivity, specificity and the determined cut-off values are calculated for the discrimination between patients with a good EULAR response (n = 4) and those with a moderate or no response (n = 14) after 4 weeks of therapy.

Receiver operating characteristic curve analysis was performed in order to assess the accuracy of tmTNF crosslinking-induced cytokines and cytokine decoy receptors as biomarkers for predicting the subsequent TNF inhibitor response. The tmTNF crosslinking-induced concentrations of IL-10, sIL1R1 and TNFR1 were all found to predict with significance the response to anti-TNF therapy. The area under the curve (AUC) was 0.9107 (95% confidence interval 0.738–1.084, p = 0.015) for IL-10, 0.9286 (95% confidence interval 0.8404–1.053, p = 0.0108) for sIL1R1 and 1.000 (95% CI 1.000–1.000; p = 0.0029) for sTNFR1. Accordingly, sTNFR1 was the biomarker with the best predictive value. The analysis identified a cut-off point of 484 pg/ml as an optimum for sTNFR1 to differentiate between patients with high and low tmTNF crosslinking-induced sTNFR1 secretion, with a sensitivity as well as specificity of 100% for the prediction of response to anti-TNF therapy. All determined cut-off values and the resulting sensitivity and specificity for the three crosslinking-induced parameters are given in detail in Fig. 5.

## Discussion

Circulating RA monocytes are known to be a heterogeneous cell population containing pathological subpopulations prone to exaggerated inflammatory responses. We report here an association of increased expression of ICAM-1 on monocytes with a less favorable response to TNF inhibition, which would imply them in the perpetuation of the autoimmune disease. The potential pathogenetic role of those cells might be the result of an increased migratory potential, therefore, which facilitates entry into the rheumatoid synovium.

In contrast, increased frequencies of TNFR1 expressing monocytes were found to be associated with a more pronounced decrease in disease activity and a better therapeutic response. One possible explanation could be, that increased surface expression of TNFR1 on monocytes is accompanied by higher concentrations of sTNFR1, which are available as decoy receptor in vivo, and which are released due to shedding of the receptor. Alternatively, increased surface expression of TNFR1 could lead to higher rates of apoptosis among pro-inflammatory monocytes due to TNFR1-TRADD signaling [13], but further studies—including the investigation of the influence of expression and activity of TACE in this scenario—are necessary to substantiate this.

TNFR1 plays a pivotal role in pro-inflammatory cytokine and chemokine secretion in RA [14]. The soluble decoy receptor sTNFR1 has been suggested to act as a TNF activity attenuator due to competition with the remaining cell-bound receptors resulting in a neutralization of TNF [15]. Indeed, knock-in mice with a non-sheddable TNFR1 mutant show an accelerated and more severe TNF-dependent chronic inflammatory arthritis [16]. Accordingly, our observation of a better therapeutic outcome in patients with monocytes releasing high levels of sTNFR1 in response to tmTNF crosslinking can also be interpreted as evidence of a possible anti-inflammatory effect of the decoy receptor, provided that sTNFR1 is released in comparable concentrations in the rheumatoid synovium in vivo.

Similarly, the induction of high levels of the anti-inflammatory cytokine IL-10 can also contribute to the therapeutic response if present in vivo. IL-10 production elicited by tmTNF reverse signaling has been reported previously [17] and interpreted as a potent anti-inflammatory response. In line with this hypothesis, we found increasing concentrations of IL-10 in sera of the patients under anti-TNF treatment, although this trend did not reach statistical significance. The significant association of IL-10 serum concentrations determined at baseline with the disease activity indicates, that up-regulation of this IL-10 secretion might indeed serve as an anti-inflammatory attenuator in the chronic autoimmune disease.

This confirms previous reports of anti-inflammatory effects of this cytokine in RA, which has been associated with less severe joint destruction [18, 19].

Concentrations of IL-8 have been reported to be elevated in sera and synovial fluid from RA patients [20], and TNF blockade is able to decrease those levels [21]. Moreover, experimental injection of IL-8 in joints of rodents leads to synovial tissue damage due to neutrophil infiltration, mimicking RA [22]. Therefore, the increased production of IL-8 in monocytes from anti-TNF treatment responders cannot readily be explained, and needs to be further investigated.

IL-1 is another monocytic cytokine involved in the inflammatory response in RA, and both soluble IL-1 receptors, sIL-1R1 [23] and sIL-1R2 [24], have been shown to act as decoy receptors. In a previous study [8], we found a negative correlation with the DAS response for concentrations of sIL-1R2 induced by reverse signaling without crosslinking, indicating that this pathway most likely does not contribute to therapeutic efficacy. In the data presented, the concentration of sIL-1R1 and sIL1-R2 elicited by tmTNF crosslinking-induced stimulation of monocytes was found to correlate significantly with the decrease of disease activity after 4 weeks of therapy. Again, this could indicate an inhibitory effect of the soluble decoy receptor, which could also occur in vivo following administration of TNF inhibiting agents.

## Conclusions

The most important result of the study is that production of the soluble decoy receptors for both TNF and IL-1 appears to be up-regulated simultaneously in patients with a good response to TNF inhibitors. Accordingly, those inhibitors of the inflammatory response could be part of a counter-regulatory mechanism that is activated in RA patients and that can be amplified by tmTNF crosslinking.

We propose, therefore, that tmTNF crosslinking due to anti-TNF therapy activates the same anti-inflammatory program, and that the responsiveness of this mechanism in vitro is a prognostic indicator of the efficacy of anti-TNF treatment in vivo.

In conclusion, we have demonstrated, that functional in vitro responses elicited in peripheral blood monocytes from RA patients by tmTNF crosslinking can be used to predict the therapeutic efficacy of anti-TNF treatment in RA. Monocytic production of cytokines and cytokine decoy receptors in particular was able to predict the response according to the EULAR criteria with a clinically meaningful sensitivity and specificity. The standardized ex vivo-analysis in patients prior to initiation of therapy could therefore be used as a diagnostic tool and provide clinically important prognostic information that can prevent primary treatment failures.

## Abbreviations

AUC: area under the curve
CCP: cyclic citrullinated peptide
CRP: C-reactive protein
DAS28: Disease Activity Score 28 joints
EULAR: European League Against Rheumatism
IL: interleukin
sIL-1R: soluble Interleukin 1 receptor
RA: rheumatoid arthritis
ROC: receiver operating characteristic
tmTNF: transmembrane tumor necrosis factor
TNF: tumor necrosis factor
TNFR1: tumor necrosis factor receptor 1
TNFR2: tumor necrosis factor receptor 2
TNFR2:Ig: tumor necrosis factor receptor 2:immunoglobulin fusion protein (Etanercept)
VAS: Visual Analog Scale
